# Inverse association between prognostic nutritional index and kidney stone prevalence: A population-based study

**DOI:** 10.1371/journal.pone.0318254

**Published:** 2025-02-18

**Authors:** Lei Wang, Yi Yu, Ziwen Jiang, Fuxiang Lin, Yuxiang Zhong, Chao Wang, Sidan Huang, Zhanping Xu

**Affiliations:** 1 The Eighth Clinical Medical College of Guangzhou University of Chinese Medicine, Foshan, Guangdong, China; 2 Foshan Maternal and Child Health Center, Foshan, Guangdong, China; 3 School of Traditional Chinese Medicine, Jinan University, Guangzhou, China; 4 School of Acupuncture-Moxibustion and Tuina, Hebei University of Chinese Medicine, Shijiazhuang, China; The First Affiliated Hospital of Soochow University, CHINA

## Abstract

**Background:**

Kidney stones frequently occur due to metabolic disorders, dietary habits, and lifestyle influences. The Prognostic Nutritional Index, which reflects an individual’s nutritional condition, might be associated with kidney stone prevalence. This study examines the association between PNI and kidney stone prevalence in US adults.

**Methods:**

The study used data from the National Health and Nutrition Examination Survey database from 2009–2018 and excluded pregnant women, and individuals who lacked data on kidney stones, or had incomplete Prognostic Nutritional Index data. Independent associations between Prognostic Nutritional Index and kidney stones were investigated by multivariate logistic regression and subgroup analyses, in addition to exploring nonlinear associations using smoothed curves and threshold effects.

**Results:**

A total of 13,835 participants aged ≥ 20 years were included, with a kidney stone prevalence of 8.48%. An inverse association was observed between the Prognostic Nutritional Index and kidney stone prevalence (OR =  0.97, 95% CI =  0.96–0.98, *P* <  0.001). This relationship was not significantly modified by race, education, marital status, or comorbidities such as hypertension, diabetes, and hyperlipidemia. However, sex and total cholesterol levels influenced the association. Stratified analysis showed a significant negative association in men (OR =  0.98, 95% CI =  0.96–0.99, *P* =  0.031), but not in women. A nonlinear relationship was identified in individuals with total cholesterol ≥  5.2 mmol/L, with a significant negative association below the inflection point of 57 (OR =  0.96, *P* =  0.012) and a positive association above it (OR =  1.11, *P* =  0.03). These findings suggest that the Prognostic Nutritional Index is inversely associated with kidney stones, particularly in men and those with high cholesterol levels.

**Conclusion:**

The Prognostic Nutritional Index was negatively associated with the risk of kidney stones, particularly in men and individuals with high cholesterol levels below the identified inflection point, suggesting that tailored nutritional management may be crucial for these subgroups.

## Introduction

Kidney stones disease is a prevalent urologic condition, affecting approximately 10–15% of the global population. In recent years, their incidence has risen significantly [1,2]. Besides posing a considerable burden on patients, kidney stones substantially strain healthcare systems worldwide. Globally, the treatment of kidney stones costs billions of dollars annually. In the United States alone, the estimated cost of treatment was $3.79 billion in 2007, projected to increase by an additional $1.24 billion annually by 2030 [1]. Moreover, kidney stones are frequently associated with complications such as urinary tract infections and renal failure, and they have a high recurrence rate [3]. Substantial evidence indicates that metabolic abnormalities, dietary habits, and lifestyle factors play critical roles in kidney stone formation [4,5]. For example, diets high in animal protein and salt, along with conditions such as obesity, diabetes, and cardiovascular disease, are linked to an increased incidence of kidney stones [6]. Thus, the prevention and management of kidney stones are crucial for affected individuals.

A growing body of research indicates that nutrition and immunity are linked to kidney stone formation [7–9]. To assess the nutritional status of patients undergoing gastrointestinal surgery, Onodera initially developed the Prognostic Nutritional Index (PNI) [10]. PNI is calculated based on serum albumin levels and the count of lymphocytes in peripheral blood. In recent years, it has been widely used for prognostic evaluation in various chronic diseases [11]. Lower PNI levels are typically linked to worse clinical outcomes, especially in individuals suffering from cardiovascular diseases, cancers, and various other health conditions [12]. The PNI has been employed to predict surgical outcomes in patients with colorectal cancer, hepatocellular carcinoma, and kidney stones [13]. Burak Arslan et al. [14] found that PNI might serve as a potential predictor for the development of SIRS/sepsis after percutaneous nephrolithotomy. Despite its use in assessing nutritional status, immune function, and predicting postoperative complications, the relationship between PNI and kidney stone prevalence has not been thoroughly studied [15,16]. Additionally, comorbidities such as cardiovascular disease may affect the association between PNI and kidney stones through metabolic pathways. It has been demonstrated that metabolic disorders, including cardiovascular disease and diabetes, may raise the risk of kidney stone formation by altering urinary pH and electrolyte balance, thereby increasing stone formation risk [6].

Accordingly, this research made use of data from the 2009–2018 NHANES to explore the relationship between PNI and kidney stone incidence, adjusting for various potential confounders.

## Materials and methods

### Data sources and study population

NHANES is an extensive cross-sectional survey using a stratified, multistage sampling method to collect information on demographics, nutritional status, and health. The dataset for this study was approved by the Ethics Review Board of the National Center for Health Statistics, under the CDC, which waived the need for additional ethical approval. Furthermore, all participants gave their informed consent in writing. For more information, refer to the relevant sections on the official CDC website (https://www.cdc.gov/nchs/nhanes/index.htm).

**Fig 1:**
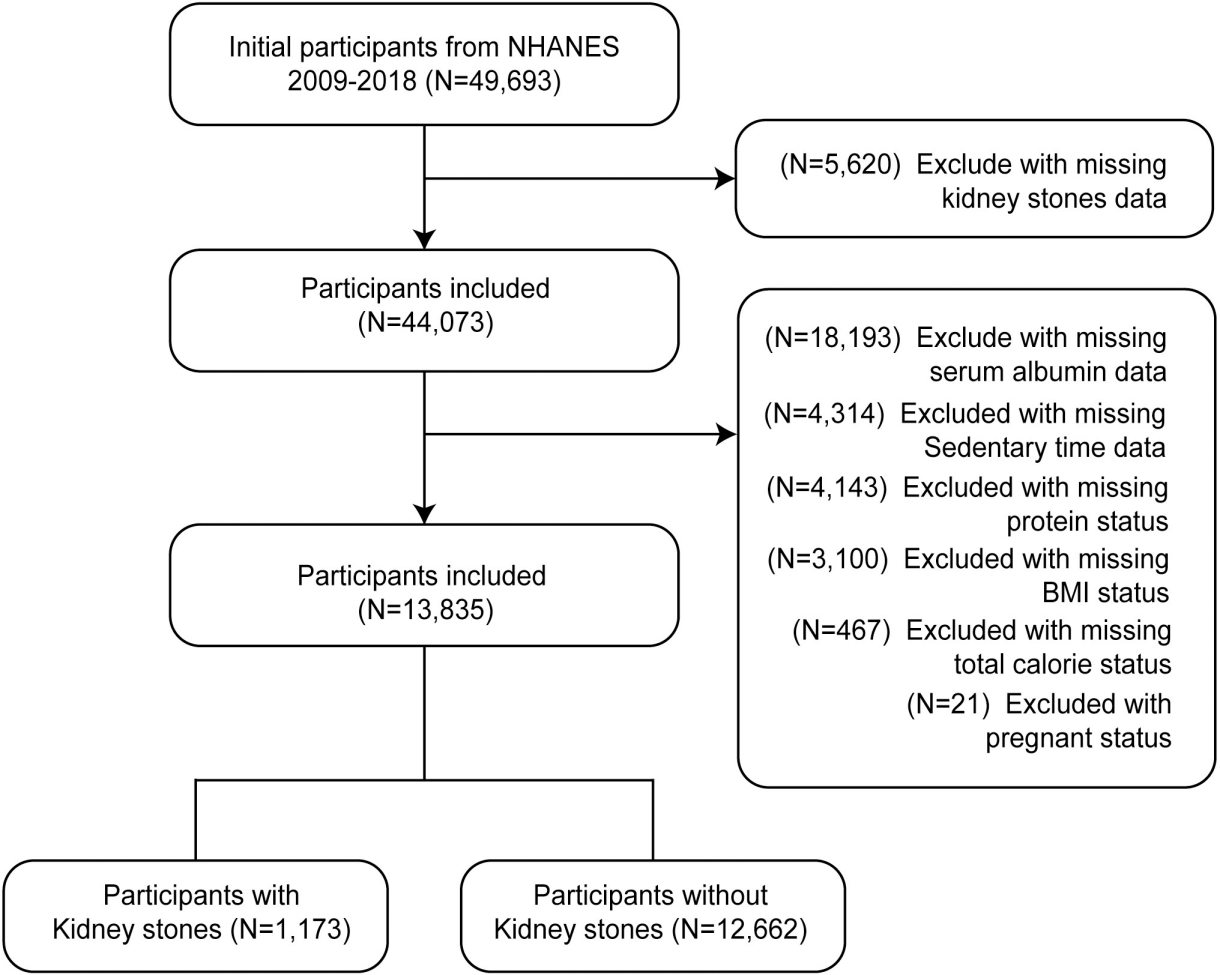
Flowchart of the selection of participants from NHANES 2009–2018.

This research utilized five cycles of NHANES data from 2009 to 2018. The study population consisted of U.S. adults aged 20 years or older, who participated in the NHANES 2009–2018 cycles and provided complete serum albumin data, serum lymphocyte count, and responses to the kidney stone history questionnaire. The inclusion criteria included: (1) age of 20 years or older, (2) availability of complete serum albumin data, serum lymphocyte count, and responses to the kidney stone history questionnaire, and (3) non-pregnant participants. A total of 49,693 individuals initially met these criteria. After excluding 18,143 participants with missing serum albumin data, 104 with incomplete lymphocyte data, and 5,620 with missing kidney stone history information, a final screening yielded 25,826 participants. Following the exclusion of 21 pregnant individuals from the covariates, 25,805 participants were ultimately included. The screening process is depicted in Fig 1.

### Exposure and outcome definition

Nutritional status was assessed using serum albumin levels measured by the bromocresol violet dye method. The PNI, used to assess a person’s nutritional condition through clinical biomarkers, is calculated by the formula [10,17,18]: PNI = (10 × serum albumin g/dL) + (0.005 × total lymphocyte counts/mm³). Serum albumin and lymphocyte counts were determined using the Beckman Coulter counting and sizing method, respectively. The diagnosis of kidney stones was based on questionnaire responses from the NHANES database, gathered through a computer-assisted personal interview system (CAPI). Professionally trained interviewers collected data at participants’ residences, focusing on their history of kidney stones. In the kidney section of the questionnaire, participants were asked, “Have you ever had a kidney stone?” Respondents who answered affirmatively were classified as having a history of kidney stones. Despite the systematic and precise nature of NHANES data collection, reliance on self-reported information introduces potential recall bias.

### Selection of covariates

To minimize bias, multiple confounders related to PNI and kidney stones were accounted for in this study. Variables considered as potential confounders included age, sex, race, educational level, PIR, marital status, smoking history, hypertension, diabetes mellitus, hyperlipidemia, coronary heart disease, sedentary time, protein intake, sodium intake, potassium intake, urea nitrogen, triglyceride, HbA1c, and eGFR. Potential confounders were selected based on their known associations with kidney stone formation as documented in the literature [19,20]. Specifically, demographic variables such as age, sex, and race were included due to their established impact on kidney stone prevalence [21]. Socioeconomic factors, including education level and PIR, were considered as they influence dietary habits and healthcare access, which in turn affect kidney stone risk [22]. Clinical conditions such as hypertension, diabetes mellitus, hyperlipidemia, and coronary heart disease were included because they are associated with metabolic abnormalities that contribute to stone formation [23]. Additionally, lifestyle factors such as sedentary time and smoking history, along with biochemical markers including serum urea nitrogen, triglyceride levels, glycosylated hemoglobin (HbA1c), eGFR, protein intake, sodium intake, and potassium intake, were included as they reflect metabolic and renal health, which are strongly linked to the pathophysiology of kidney stones [20].

Three demographic variables were included in the partially adjusted models: gender, age, and race. Racial categories were classified as non-Hispanic white, non-Hispanic black, Hispanic/Latino, and other races. In the fully adjusted model, additional covariates were considered, including education level, marital status, the ratio of family income to poverty (PIR), body mass index (BMI), hypertension, diabetes mellitus, hyperlipidemia, coronary heart disease, smoking history, sedentary time (minutes/day), serum urea nitrogen (mg/dL), triglycerides (mmol/L), glycosylated hemoglobin (HbA1c, %), total cholesterol (mmol/L), estimated glomerular filtration rate (eGFR; mL/[min/1.73 m²]), protein intake (mg/day), sodium intake (mg/day), and potassium intake (mg/day). Education level was classified into three groups: below high school, high school, and above high school. Poverty-to-income ratios were calculated by dividing household income by the Census Bureau’s annual poverty line. Marital status was classified as married/cohabiting, widowed, divorced, separated, and unmarried. Hypertension, diabetes mellitus, hyperlipidemia, and coronary heart disease were identified based on participants’ responses to whether they had ever been diagnosed by a doctor with these conditions. If the response was “yes,” the condition was considered present.

Smoking history was determined by asking, “Have you ever smoked at least 100 cigarettes in your lifetime?” A “yes” response indicated a smoking history. Sedentary time was recorded in minutes per day based on participants’ self-reported daily sedentary activity. Protein, sodium, and potassium intake were calculated based on dietary data collected through a 24-hour recall. Values for serum urea nitrogen, triglycerides, glycosylated hemoglobin, and total cholesterol were obtained from laboratory results. The eGFR (mL/[min/1.73 m²]) was calculated using the CKD-EPI equation [24].

### Statistical analysis

Statistical analyses were performed according to CDC guidelines. Continuous variables were summarized as means with standard deviations or medians with interquartile ranges, while categorical variables were shown as percentages. Group comparisons were conducted using the chi-square test for categorical variables, the Kruskal-Wallis test for non-normally distributed continuous variables, and ANOVA for normally distributed continuous variables. Logistic regression models were used to compute odds ratios (OR) with 95% confidence intervals (CI) to adjust for confounders and evaluate the association between PNI and kidney stone prevalence. Clinically significant factors from previous studies were included as covariates to minimize confounding effects [19,25].

Three logistic regression models were developed. Model 1 was unadjusted; Model 2 adjusted for demographic factors (gender, age, race); and Model 3 included additional variables such as education, marital status, poverty-to-income ratio (PIR), Body mass index (BMI), sedentary time, hypertension, diabetes, hyperlipidemia, coronary artery disease, smoking history, urea nitrogen, triglycerides, glycosylated hemoglobin, total cholesterol, eGFR, Protein intake, sodium intake and potassium intake. PNI values were also categorized into quartiles to explore the association across different levels, and stratified logistic regression was conducted to assess subgroup-specific associations.

Nonlinear relationships between PNI and kidney stone prevalence were analyzed using smoothed curve fitting via a generalized additive model (GAM). Inflection points were identified using a two-segment linear regression model, with significance determined via the Log-likelihood ratio test. Local variations in the curves were considered non-significant. Sampling weights were not applied in multivariable analyses to avoid over-adjustment bias, as some covariates used in NHANES weighting calculations were included in the regression models. Descriptive statistics were calculated without applying survey weights, with further implications discussed in the “Discussion” section. Statistical analyses were conducted using R software (version 4.0.5; http://www.R-project.org) and EmpowerStats (version 2.0; http://www.empowerstats.com).

## Results

### Characteristics

Between 2009 and 2018, a total of 13,835 U.S. adults participated in the survey, including 6,584 men (47.59%) and 7,251 women (52.41%). Among them, 1,173 participants (8.48%) were classified as former kidney stone patients, while 12,662 (91.52%) were classified as non-kidney stone participants. The average age of the participants was 49.37 ±  17.67 years, with the kidney stone group being older (55.90 ±  16.18 years) compared to the non-kidney stone group (48.76 ±  17.68 years). Racial demographics showed that non-Hispanic whites had the highest prevalence of kidney stones (52.94%), while non-Hispanic blacks had the lowest prevalence (12.87%). Significant differences between the kidney stone and non-kidney stone groups were observed in variables such as age, sex, race, marital status, hypertension, diabetes mellitus, hyperlipidemia, coronary heart disease, smoking history, BMI, sodium intake, sedentary time, urea nitrogen, triglycerides, HbA1c, eGFR, and PNI (P <  0.05). The mean PNI value was lower in the kidney stone group (52.15 ±  5.21) compared to the non-kidney stone group (53.15 ±  5.21). Sodium intake was slightly higher in the kidney stone group (3270.82 ±  1415.96 mg/day), while sedentary time was lower (332.64 ±  190.64 minutes/day). Other factors such as potassium and protein intake showed no significant differences between the groups. The mean PNI value in the study population was 53.1 ±  13.2, ranging from a minimum of 35.2 to a maximum of 85.5 (see Table 1). This range indicates that a ‘1-point change in PNI’ represents a meaningful and feasible variation within the population.

**Table 1:** Baseline characteristics of the study participants.

Characteristics	Total	Kidney stone	Non-Kidney stone	*P*-value
	N = 13835	N = 1173	N = 12662	
**Age (years)**	49.37 ± 17.67	55.90 ± 16.18	48.76 ± 17.68	<0.001
**Sex (n/%)**				<0.001
Male	6584 (47.59)	627 (53.45)	5957 (47.05)	
Female	13304 (52.41)	546 (46.55)	6705 (52.95)	
**Race (n/%)**				<0.001
Non-Hispanic White	5433 (39.27)	621 (52.94)	4812 (38.00)	
Non-Hispanic Black	2912 (21.05)	151 (12.87)	2761 (21.81)	
Hispanic/Latino	1466 (10.60)	131 (11.17)	1335 (10.54)	
Non-Hispanic other	4024 (29.09)	270 (23.02)	3754 (29.65)	
**Education (n/%)**				0.552
Less than high school	1400 (10.12)	129 (11.00)	1271 (10.04)	
High school or GED	1852 (13.39)	159 (13.55)	1693 (13.37)	
Above high school	10583 (76.49)	885 (75.45)	9698 (76.59)	
**Marital status (n/%)**				<0.001
Married/Living with a partner	8147 (58.89)	739 (63.00)	7408 (58.51)	
Divorced/Widowed/Separated	3075 (22.23)	316 (26.94)	2759 (21.79)	
Never married	2613 (18.89)	118 (10.06)	2495 (19.70)	
**Hypertension (n/%)**				<0.001
Yes	4965 (35.89)	590 (50.30)	4375 (34.55)	
No	8870 (64.11)	583 (49.70)	8287 (65.45)	
**Diabetes mellitus (n/%)**				<0.001
Yes	1835 (13.26)	268 (22.85)	1567 (12.38)	
No	12000 (86.74)	905 (77.15)	905 (77.15)	
**Hyperlipidemia (n/%)**				<0.001
Yes	6642 (48.01)	565 (48.17)	6077 (47.99)	
No	3088 (22.32)	159 (13.55)	2929 (23.13)	
Unclear	4105 (29.67)	449 (38.28)	3656 (28.87)	
**Coronary heart disease (n/%)**				<0.001
Yes	540 (3.90)	90 (7.67)	5291 (41.79)	
No	13295 (96.10)	1083 (92.33)	12212 (96.45)	
**Smoking history (n/%)**				<0.001
Yes	5899 (42.64)	608 (51.83)	5291 (41.79)	
No	7936 (57.36)	565 (48.17)	7371 (58.21)	
**BMI**	28.81 ± 7.37	29.71 ± 7.52	28.72 ± 7.35	<0.001
**PIR**	2.48 ± 1.63	2.49 ± 1.54	2.48 ± 1.55	0.967
**Sodium intake (mg)**	3357.14 ± 1440.78	3270.82 ± 1415.96	3365.17 ± 1442.85	0.002
**Potassium intake (mg)**	2483.03 ± 1036.85	2488.75 ± 1015.09	2482.50 ± 1038.88	0.362
**Protein intake (mg)**	78.86 ± 42.47	76.00 ± 37.85	79.13 ± 42.86	0.125
**Sedentary time (minutes)**	387.78 ± 200.68	332.64 ± 190.64	403.82 ± 200.53	<0.001
**Urea nitrogen (mg/dL)**	14.0 ± 6.1	15.37 ± 7.61	13.71 ± 5.84	<0.001
**Triglyceride (mmol/L)**	1.8 ± 1.4	1.85 ± 1.59	1.70 ± 1.32	<0.001
**HbA1c/%**	5.8 ± 1.1	6.02 ± 1.24	5.77 ± 1.06	<0.001
**TC (mmol/L)**	5.0 ± 1.1	4.91 ± 1.15	4.95 ± 1.07	0.226
**eGFR (mL/[min 1.73 m** ^**2**^ **])**	95.9 ± 24.6	88.35 ± 24.55	97.02 ± 24.21	<0.001
**PNI**	52.8 ± 4.8	52.05 ± 5.06	52.98 ± 4.82	<0.001

Note: Values are expressed as weighted means ±  SE or %.

Abbreviation: PNI, Prognostic nutritional index; PIR, the ratio of family income to poverty; TC, Total cholesterol; HbA1c, Glycosylated hemoglobin; eGFR, Estimated Glomerular filtration rate; BMI, Body mass index.

### Logistic regression analysis of kidney stone prevalence

The results showed that higher PNI levels were significantly linked to a lower prevalence of kidney stones. In the unadjusted model (Model 1), each 1-unit increase in PNI was associated with a 4% decrease in kidney stone prevalence (OR =  0.96; 95% CI =  0.95–0.97; *P* <  0.001). After adjusting for demographic variables in Model 2, each 1-unit rise in PNI led to a 2% decrease in risk (OR =  0.98; 95% CI =  0.96–0.99; *P* =  0.002). In Model 3, after further adjusting for covariates, every 1-unit increase in PNI corresponded to a 1% reduction in kidney stone prevalence (OR =  0.99; 95% CI =  0.97–1.00; *P* =  0.030). The relationship was consistent across all models.

Additionally, the relationship was validated by converting PNI into quartiles for stratified analysis. In the fully adjusted model, individuals in the highest PNI quartile (Q4) exhibited a 21% reduced prevalence of kidney stones compared to those in the lowest quartile (Q1) (Model 3: OR =  0.79; 95% CI =  0.66–0.96; *P* =  0.014). This pattern was consistent across all models, with trend test P-values below 0.05 (refer to Table 2).

**Table 2:** Multivariate logistic regression to assess the association between PNI and kidney stones.

Variable	Model 1		Model 2		Model 3	
	OR(95% CI)	*P-value*	OR(95% CI)	*P-value*	OR(95% CI)	*P-value*
PNI	0.96(0.95, 0.97)	<0.001	0.98(0.96, 0.99)	0.002	0.99 (0.97, 1.00)	0.030
Q1	Ref		Ref		Ref	
Q2	0.86 (0.74, 1.02)	0.077	0.93 (0.79, 1.10)	0.399	0.98 (0.83, 0.17)	0.859
Q3	0.71 (0.60, 0.84)	<0.001	0.82 (0.69, 0.97)	0.038	0.91 (0.76, 1.08)	0.277
Q4	0.65 (0.50, 0.70)	<0.001	0.71 (0.95, 0.98)	0.001	0.79(0.66, 0.96)	0.014
*P*-for trend		<0.001		<0.001		0.011

In sensitivity analysis, PNI was converted from a continuous variable to a categorical variable (quartile).

Model 1 =  No covariates were adjusted. Model 2 =  Age, gender, and race were adjusted. Model 3 =  Age, gender, race, educational level, BMI, Sedentary time, Marital status, PIR, Hypertension, Diabetes mellitus, Hyperlipidemia, Coronary heart disease, Smoking history, Urea nitrogen, Triglyceride, HbA1c, TC, eGFR, Protein intake, Sodium intake, and Potassium intake were adjusted.

Abbreviation: OR, Odds ratio; 95% CI, 95% confidence interval; PNI, Prognostic nutritional index; PIR, the ratio of family income to poverty; TC, Total cholesterol; HbA1c, Glycosylated hemoglobin; eGFR, Estimated Glomerular filtration rate; BMI, Body mass index.

### Subgroup analysis

**Fig 2:**
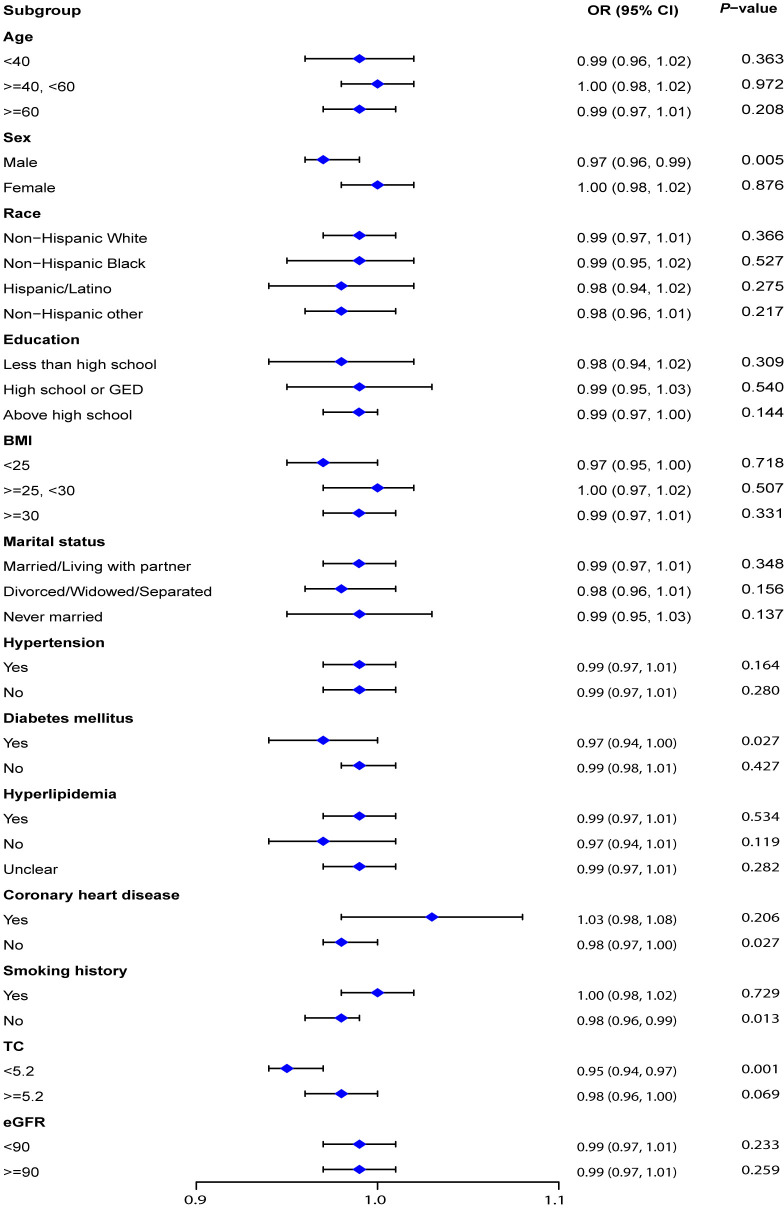
Subgroup analysis of the association between PNI and kidney stones. Abbreviation: PNI, Prognostic nutritional index; PIR, the ratio of family income to poverty; TC, Total cholesterol; HbA1c, Glycosylated hemoglobin; eGFR, Estimated Glomerular filtration rate; BMI, Body mass index.

As shown in Fig 2, sex and total cholesterol modified the relationship between PNI and the likelihood of kidney stone prevalence. PNI was significantly associated with a lower prevalence of kidney stones in men (OR:0.98; 95% CI: 0.96–0.99; *P* trend =  0.031), with no significant association observed in women. Similarly, the negative association between PNI and kidney stones was evident in individuals with low total cholesterol (OR: 0.98; 95% CI: 0.96–0.99; *P* trend =  0.017), but not in those with high total cholesterol. The other covariates did not have a significant impact on the connection between PNI and kidney stones (*P* > 0.05). These results indicate that the link between PNI and kidney stones is strong and remains largely unaffected by most confounders.

### Smooth curve fitting and threshold effect analysis

**Fig 3:**
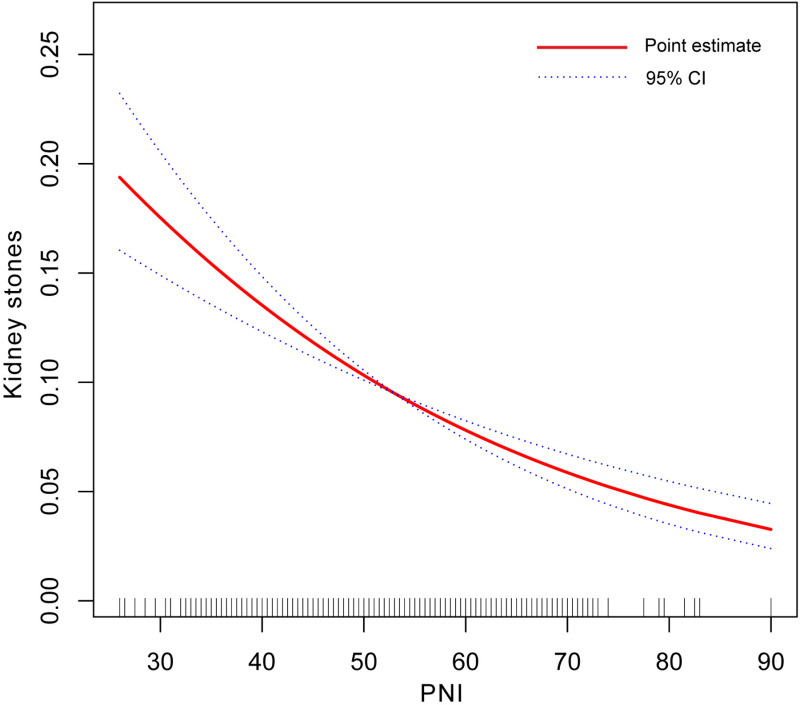
A linear relationship between PNI and kidney stones by the generalized additive model. Age, gender, race, educational level, BMI, Sedentary time, Marital status, PIR, Hypertension, Diabetes mellitus, Hyperlipidemia, Coronary heart disease, Smoking history, Urea nitrogen, Triglyceride, HbA1c, TC, eGFR, Protein intake, Sodium intake, and Potassium intake were adjusted. Abbreviation: OR, Odds ratio; 95% CI, 95% confidence interval; PNI, Prognostic nutritional index; PIR, the ratio of family income to poverty; TC, Total cholesterol; HbA1c, Glycosylated hemoglobin; eGFR, Estimated Glomerular filtration rate; BMI, Body mass index.

**Fig 4:**
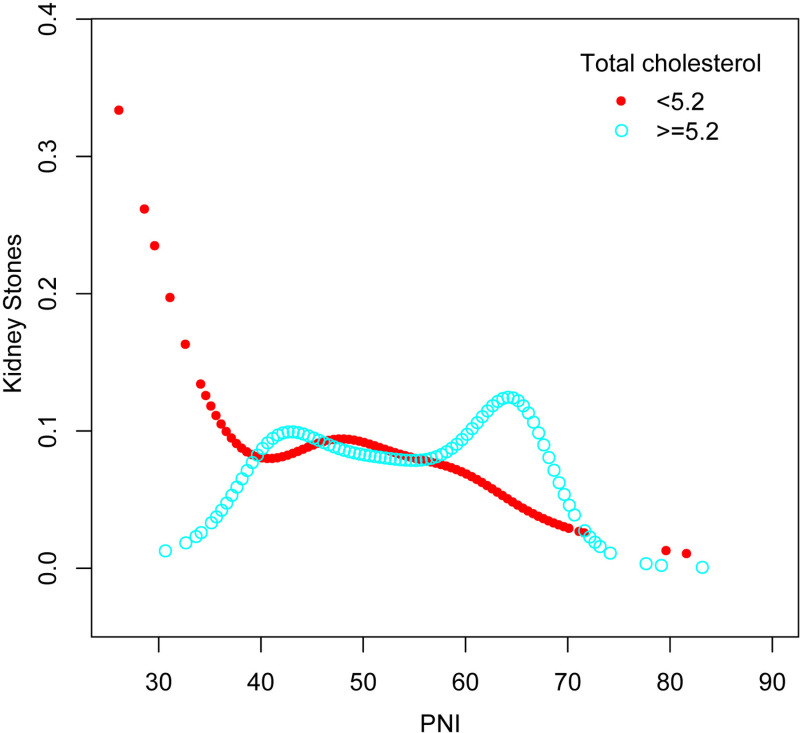
Adjusted dose-response relationship between PNI and kidney stone incidence stratified by total cholesterol levels. Age, gender, race, educational level, BMI, Sedentary time, Marital status, PIR, Hypertension, Diabetes mellitus, Hyperlipidemia, Coronary heart disease, Smoking history, Urea nitrogen, Triglyceride, HbA1c, eGFR, Protein intake, Sodium intake, and Potassium intake were adjusted.

**Fig 5:**
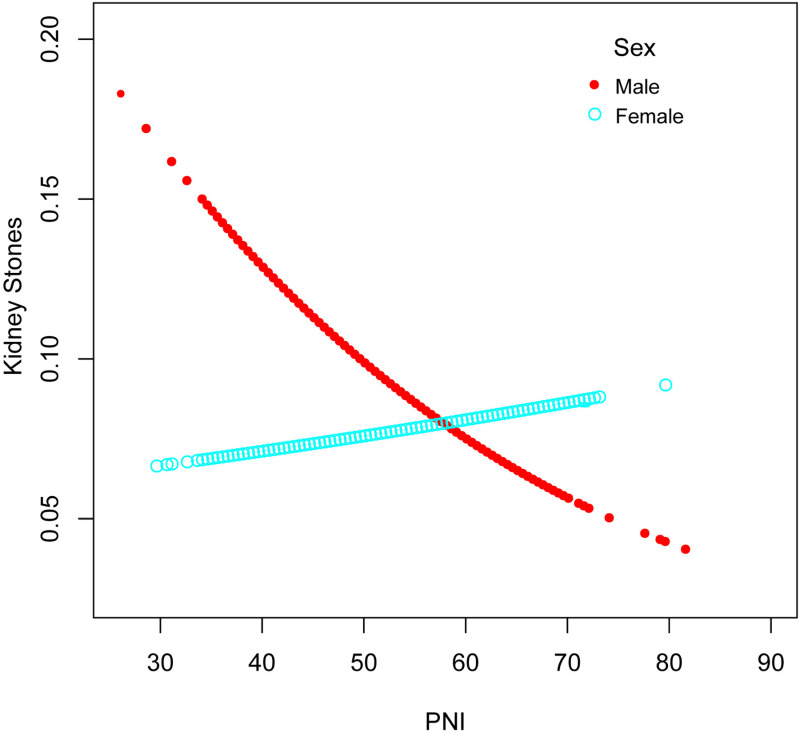
Adjusted dose-response relationship between PNI and kidney stone incidence stratified by sex. Age, race, educational level, BMI, Sedentary time, Marital status, PIR, Hypertension, Diabetes mellitus, Hyperlipidemia, Coronary heart disease, Smoking history, Urea nitrogen, Triglyceride, HbA1c, TC, eGFR, Protein intake, Sodium intake, and Potassium intake were adjusted.

The results revealed a nonlinear relationship between PNI and the prevalence of kidney stones, as observed in Fig 3. Stratified analysis by total cholesterol levels and sex identified a significant turning point for total cholesterol (Table 3 and Fig 4). Among participants with total cholesterol levels ≥ 5.2 mmol/L, the inflection point occurred at a PNI value of 57 (*P* for Log-likelihood ratio =  0.001). Below this threshold, PNI was negatively associated with kidney stone prevalence (OR =  0.96; 95% CI: 0.94–1.00; *P* =  0.012), while above this value, the association reversed, becoming positive (OR =  1.11; 95% CI: 1.04–1.19; *P* =  0.03). In contrast, no significant association between PNI and kidney stone prevalence was observed in participants with total cholesterol levels < 5.2 mmol/L (*P* >  0.05). When stratified by sex (Fig 5), PNI consistently showed a negative association with kidney stone prevalence in men (OR =  0.98; 95% CI: 0.96–0.99;). However, in women, while a positive association was observed, it did not reach statistical significance. These findings highlight the differential impact of PNI on kidney stone prevalence based on cholesterol levels and sex.

**Table 3:** Threshold effect analysis of kidney stones using a two-segment linear regression model.

Variable	OR	95% CI	*P*-value
TC (﹤5.2mmol/L)			
PNI	0.98	(0.97, 1.00)	0.040
Inflection point			
<51.5	1.00	(0.97, 1.04)	0.762
>51.5	0.96	(0.93,0.99)	0.012
*P* for Log-likelihood ratio test			0.085
TC ( ≥ 5.2mmol/L)			
PNI	0.99	(0.97, 1.02)	0.589
Inflection point			
<57	0.96	(0.94, 1.00)	0.012
>57	1.11	(1.04, 1.19)	0.003
*P* for Log-likelihood ratio test			0.001

Age, gender, race, educational level, BMI, Sedentary time, Marital status, PIR, Hypertension, Diabetes mellitus, Hyperlipidemia, Coronary heart disease, Smoking history, Urea nitrogen, Triglyceride, HbA1c, TC, eGFR, Protein intake, Sodium intake, and Potassium intake were adjusted.

OR, Odds ratio; 95% CI, 95% confidence interval; PNI, Prognostic nutritional index; PIR, the ratio of family income to poverty; TC, Total cholesterol; HbA1c, Glycosylated hemoglobin; eGFR, Estimated Glomerular filtration rate; BMI, Body mass index.

The inflection points in both Figs 4 and 5 were identified using a two-segment linear regression model, with statistical significance supported by the Log-likelihood ratio test. The curves represent smoothed estimates from a generalized additive model (GAM), illustrating the nonlinear relationship between PNI and kidney stone prevalence.

## Discussion

This study is the first to demonstrate a significant inverse association between PNI and the risk of kidney stone prevalence, based on an analysis of data from 25,805 participants (OR =  0.97, *P* <  0.001). This relationship held steady after accounting for various confounding factors, (*P* > 0.05). A nonlinear association between PNI and kidney stone prevalence was detected. Generalized additive models and segmented regression analyses identified a threshold effect, particularly in participants with total cholesterol levels below 5.2 mmol/L. In this group, a significant threshold effect was evident, with a PNI value of 61.5 serving as the inflection point. Below this value, the risk of kidney stones was markedly reduced (OR =  0.85).

To our knowledge, this is the first study to examine the relationship between PNI and kidney stone prevalence, highlighting the link between higher PNI levels and a reduced prevalence of kidney stones. PNI incorporates serum albumin and lymphocyte levels as key components [26,27]. Both are closely associated with nutritional status: serum albumin reflects protein-energy malnutrition, as hepatic albumin synthesis depends on adequate protein intake, while lymphocyte levels are indicative of immune function, which can be impaired by malnutrition [28,29]. Nutritional deficiencies lead to reduced albumin levels and impaired immune function, decreasing lymphocyte counts and potentially increasing the risk of kidney stones [30].

Lee HY et al. evaluated the recurrence rate of urolithiasis using the Controlling Nutritional Status (CONUT) score and found a significant negative correlation between nutritional status and kidney stone recurrence (OR =  1.736, 95% CI =  1.041–2.896, *P* =  0.034). They also observed that recurrence occurred more rapidly in patients with poorer nutritional status (log-rank test, *P* =  0.014) [31,32]. While the findings align with our study, PNI appears to be a more sensitive clinical prognostic indicator compared to CONUT, supporting the rationale for our investigation [26].

Our study identified a significant inverse association between PNI and kidney stone prevalence, suggesting that nutritional and immune status plays a crucial role in kidney stone formation. The mechanisms underlying this association can be explained by the roles of serum albumin and lymphocytes。

Serum albumin is a marker of long-term nutritional reserves, with a half-life of approximately 21 days [33]. Beyond reflecting protein-energy nutritional status, albumin possesses antioxidant, anti-inflammatory, and calcium ion-binding functions. Low albumin levels increase oxidative stress, a major contributor to kidney stone formation [34]. Additionally, low albumin levels are associated with chronic inflammation, which promotes stone formation by exacerbating renal tissue damage [35,36]. Albumin also binds calcium ions to maintain urinary calcium homeostasis. A reduction in albumin levels can lead to increased urinary calcium concentrations, promoting the formation of calcium-based stones [37,38].

Lymphocytes broadly reflect immune function, even though they can fluctuate under acute stress. A decrease in lymphocyte levels weakens immune defenses, increasing the risk of urinary tract infections, a major trigger for kidney stones [39]. Calcium oxalate crystals stimulate lymphocytes to release pro-inflammatory cytokines, such as IL-1β and IL-18, which exacerbate renal inflammation through the NLRP3 inflammasome pathway [40,41]. Additionally, lymphocyte-regulated proteins like CD44 promote the adhesion of crystals to renal epithelial cells, facilitating stone formation [42,43].

Our subgroup analysis demonstrated that the association between PNI and kidney stone prevalence was more pronounced in men and individuals with low cholesterol levels. Yao et al. reported a significant association between elevated residual cholesterol and kidney stone formation [20]. Similarly, Liu et al. found a strong inverse association between cholesterol levels and kidney stones among patients with metabolic syndrome (OR =  0.66, 95% CI =  0.55–0.79) [5]. Metabolic syndrome, characterized by abnormal urinary metabolism of calcium, oxalate, and uric acid, may explain the increased risk of kidney stones in these individuals [44]. Furthermore, men are more prone to metabolic imbalances, such as hyperuricemia and hypercalciuria, due to higher metabolic rates, which may account for the stronger association between PNI and kidney stones in this subgroup [45,46].

The following are the benefits of our study. First, by using nutritional measures to lower the risk of kidney stones, PNI, a straightforward and commonly available assessment tool, may assist in the early identification of high-risk patients. Second, this study shows a nonlinear association between PNI and kidney stone prevalence for the first time, suggesting that PNI may be a useful predictor of kidney stone prevalence, particularly in high-risk patients. Furthermore, the study’s representative sample and large sample size enhanced the robustness of the findings.

However, because of the study’s cross-sectional nature, it was not possible to establish a causal link between PNI and the risk of kidney stones, despite the observed association. Additionally, a key limitation is the decision not to apply sampling weights in multivariable analyses. While weighting is standard practice to ensure population-level generalizability, it may introduce over-adjustment bias when key covariates are also part of the weighting calculations. Our approach focused on analyzing relative associations and trends rather than estimating population-representative prevalence. We acknowledge this may limit the generalizability of our findings, and future studies should explore weighted analyses to validate and expand these results. To better establish the causative role of PNI in kidney stone formation, future studies should adopt a longitudinal approach.

## Conclusion

The current investigation demonstrated a substantial negative correlation between the occurrence of kidney stones and the PNI, particularly in the male and high-cholesterol population. All things considered, PNI may play a significant role in kidney stone prediction and prevention as an indicator of immunological and nutritional function, offering fresh insights into customized nutritional management plans.
